# Pathogenetic characterization of a *Micrococcus luteus* strain isolated from an infant

**DOI:** 10.3389/fped.2023.1303040

**Published:** 2023-12-22

**Authors:** Xiaolu Shi, Shuxiang Qiu, Liyin Ji, Huiqun Lu, Shuang Wu, Qiongcheng Chen, Xuan Zou, Qinghua Hu, Tiejian Feng, Shiting Chen, Wenkai Cui, Shiqin Xu, Min Jiang, Rui Cai, Yijie Geng, Qinqin Bai, Dingjie Huang, Peihui Liu

**Affiliations:** ^1^ Department of Public Health Laboratory Sciences, School of Public Health, Hengyang Medical School, University of South China, Hengyang, China; ^2^ Shenzhen Center for Disease Control and Prevention, Shenzhen, China; ^3^ Shenzhen Research Center for Communicable Disease Control and Prevention, Chinese Academy of Medical Sciences, Shenzhen, China; ^4^ School of Public Health, Shanxi Medical University, Taiyuan, China; ^5^ Pediatric Department, Shenzhen Maternity and Child Healthcare Hospital, Shenzhen, China

**Keywords:** *Micrococcus luteus*, bloodstream infection, pathogenesis, genomics, virulence

## Abstract

**Purpose:**

To explore the clinical characteristics of *Micrococcus luteus* bloodstream infection in an infant and characterize the phenotype and genotype of the isolated strains, as well as seek suitable infection models for assessing virulence.

**Methods:**

Clinical data was collected from an infant patient diagnosed with *M. luteus* bloodstream infection. Metagenomic sequencing was performed on the isolated blood sample. The strain was isolated and underwent mass spectrometry, biochemical tests, antibiotic susceptibility assays, and whole-genome sequencing. The *Galleria mellonella* infection model was used to assess *M. luteus* virulence.

**Results:**

Patient responded poorly to cephalosporins, but recovered after Linezolid treatment. Metagenomic sequencing identified *M. luteus* as the predominant species in the sample, confirming infection. They were identified as *M. luteus* with a high confidence level of 98.99% using mass spectrometry. The strain showed positive results for Catalase, Oxidase, and Urea tests, and negative results for Mannose, Xylose, Lactose, Mannitol, Arginine, and Galactose tests, consistent with the biochemical profile of *M. luteus* reference standards. *M. luteus* susceptibility to 15 antibiotics was demonstrated and no resistance genes were detected. Predicted virulence genes, including *clpB*, were associated with metabolic pathways and the type VI secretion system. The infection model demonstrated dose-dependent survival rates.

**Conclusion:**

The infant likely developed a bloodstream infection with *M. luteus* due to compromised immunity. Although the isolated strain is sensitive to cephalosporin antibiotics and has low pathogenicity in infection models, clinical treatment with cephalosporins was ineffective. Linezolid proved to be effective, providing valuable guidance for future clinical management of such rare infections.

## 1. Introduction

*Micrococcus luteus*, a Gram-positive coccus of the genus *Micrococcaceae*, is widely distributed in the environment, including in soil, air, water and on animals (1, 2). *M. luteus* is rarely reported in clinical cases (3) though, as an opportunistic pathogen (4), it has been reported to cause disease in susceptible patients, such as those with malnutrition and poor immunity (5, 6). For example, Albertson et al. (7) reported septic shock caused by *M. luteus* in a patient with valveless heart disease and an implanted prosthesis. Buonsenso et al. (8) reported natural valvular complications of pulmonary infarction in a pediatric patient resulting from *M. luteus* infection. Although reports of human infection with *M. luteus* are rare, determining potential antibiotic susceptibility and virulence of *M. luteus* may ensure effective treatment of people susceptible to infection.

*Galleria mellonella* has no ethical constraints and a short life cycle (9). The *G. mellonella* immune system exhibits both humoral and cellular components, and in some aspects the immune response is similar to the innate immune response of mammals (10). As such, *G. mellonella* larvae have been successfully utilized as a model organism in a variety of bacterial infection experiments (11). In addition, there is no reports indicating animal infection model of *M. luteus*.

In this study we identified a case of bloodstream infection, analyzed the patient's clinical treatment, identified the pathogen, and explored its pathogenicity to provide information about this rare infection. The virulence of the pathogen was evaluated by using a model organism of *M. luteus* infecting *G. mellonella* larvae, providing a basis for studying the pathogenesis of *M. luteus*.

## 2. Materials and methods

### 2.1. Collecting case information

This study involved the collection of patient information from an individual who exhibited recurrent fever symptoms and had two consecutive positive blood cultures for *M. luteus*. The gathered data encompassed age, gender, results of laboratory tests, administered treatments, and clinical outcome.

### 2.2. Pathogen identification

#### 2.2.1. Isolation culture and staining

A sample of the patient's blood was inoculated onto a plate containing Columbia blood agar medium (Guangzhou Detgerm Microbiogical Science Ltd, Guangdong, China) and kept at a constant temperature of 37°C for a 48 h incubation. After incubation, individual colonies with a consistent appearance were picked for culturing. A Gram staining kit (Huankai Microbial, Guangdong, China) was used to stain bacteria for microscopic observation using a Zeiss fully automatic positive fluorescence microscope Axio Imager M2 (ZEISS, Oberkochen, Germany) (12).

#### 2.2.2. Mass spectrometry identification

Using the direct coating method, *Escherichia coli* ATCC8739 was used as the calibration strain, and a small amount of pure, cultured individual colonies were evenly coated with 1μl inoculation loop to target plate spots. Immediately, 1 ul of alpha-cyano-4-hydroxy-cinnamic acid (CHCA) substrate was added and, when the substrate was completely dry, strain identification occurred using the standard operating procedure of Matrix-Assisted Laser Desorption/Ionization Time of Flight Mass Spectrometry (MALDI-TOF/MS; BioMerieux, Lyon, France)**.**

#### 2.2.3. Physiological and biochemical identification

A homogenous bacterial suspension, equivalent to a turbidity of 0.50–0.63 McFarland, was prepared by calibrating the turbidimeter (BioMerieux, Lyon, France) with a sterile swab and inoculating identical colonies into prepared sterile saline tubes. The bacterial suspension tubes and Gram-positive bacterial identification cards (Biomerieux, Lyon, France) were placed in the card rack of the VITEK 2 Compact Automatic Microbiological Identification and Drug Sensitivity Analyzer and operated according to the guidelines. The Gram-positive bacterial identification cards include multiple biochemical details such as peroxidase, oxidase, mannose, xylose, lactose, mannitol, urea, galactose, and others. The bacterial identification results were referred to the *Bergey’s Manual of Determinative Bacteriology* (13).

#### 2.2.4. Antibiotic susceptibility test

The broth microdilution method (Fosun Diagnostics, Shanghai, China) was used to test the antibiotic sensitivity of *M. luteus* strains. Penicillin, Vancomycin, Erythromycin, Clindamycin, Moxifloxacin, Cefotaxime, Tetracycline, Linezolid, Cotrimoxazole, Meropenem, Chloramphenicol, Amoxicillin, Levofloxacin, Teicoplanin, and Cefepime were tested. Breakpoints for sensitive (S), intermediate (I), and resistant (R) were defined by the Clinical and Laboratory Standards Institute (CLSI) document M45 (http://www.clsi.org), and *Staphylococcus aureus* ATCC 29213 was used as the control.

#### 2.2.5. Bacterial genomic DNA extraction and whole genome sequencing

The total DNA of Gram-positive bacteria was extracted using the Ezup column-based bacterial DNA extraction kit (Sangon Biotech, Shanghai, China), and the quality of nucleic acids was confirmed using a NanoDrop one ultra-micro spectrophotometer (Thermo Fisher Scientific, Massachusetts, America). Small fragment libraries, with an average insert size of 350 bp, were prepared by Beijing Novozymes Technology Co. using the Illumina NovaSeq 6000 platform. Sequencing evaluation and screening were then performed. Trimmomatic software (v0.39) (14) was used to quality-control filter the raw data and obtain valid data, and SPAdes gene assembly software (V3.9.1) (15) was used to splice and assemble the valid sequences. Kraken2 software (16) was used to identify the possible pathogens for the valid sequences after successful splicing. Investigation of carriage of antibiotic resistance and virulence genes was assessed comprehensively using the ResFinder7 database (17), the CARD database (18) and the VFDB6 database (19). Chiplot (https://www.chiplot.online/) was used to visualize the gene density, gene function annotation, and CG content of the whole bacterial genome.

#### 2.2.6. Nucleic acid extraction of blood pathogens and sequencing of microbial genomes

After blood cultures were established as positive for Gram-positive cocci, pathogenic DNA was extracted from blood samples using the QIAamp DNA Blood Mini Kit by (QIAGEN, Guangdong, China). Metagenomic sequencing was performed by Novogene (Beijing, China). Using Readfq (v8) (http://github.com/lh3/readfq), preprocess raw data was obtained from the Illumina HiSeq sequencing platform for subsequent analysis (Clean Data). Bowtie2 software (v2.2.4) (20) was used to delete host readings and MEGAHIT software (v1.0.4 beta) (21) was used to assemble and analyze Clean Data. DIAMOND (v0.9.9.110) (22) was used to integrate unigenes with the National Center for Biotechnology Information (NCBI) non-redundant (NR) database (https://www.ncbi.nlm.nih.gov/), compare bacteria, fungi, archaea, and viruses, and annotate species information using Lowest Common Ancestor (LCA) algorithm on January 18, 2018. Short-read sequencing data from isolated samples have been deposited in the NCBI Sequence Read Archive under the BioProject PRJNA973819.

#### 2.2.7. *Galleria mellonella* infection model

The isolated strain of *M. luteus* was inoculated into a blood plate, and incubated at 37°C for 48 h. Single bacterial colonies were then placed into 2 ml of nutrient broth (Huankai Microbial, Guangdong, China) for 12 h then centrifuged at 8,000 r/min for 3 min and washed three times with physiological saline. Bacteria were resuspended in 1 ml of physiological saline and the following bacterial suspension doses were prepared: 5 × 10^2 ^CFU/ml (low dose), 5 × 10^4 ^CFU/ml (medium dose) and 5 × 10^6^ CFU/ml (high dose). *G. mellonella* larvae (250–350 mg/piece) were fasted for 24 h then 10 larvae per dose group (high, medium, and low) were injected with 10 μl of the corresponding bacterial suspension using a 10 μl micro syringe. Three forms of control were set: a blank control group of 10 larvae (without any treatment), a puncture group of 10 larvae (with a micro syringe puncture without injection of physiological saline), and a physiological saline group of 10 larvae (injected with 10 μl of physiological saline). *G. mellonella* larvae were incubated at 37°C for 72 h after which surface color changes, response to stimuli, and survival of the larvae were observed every 24 h. *G. mellonella* were considered dead when unresponsive to external stimuli (23–26).

### 2.3. Statistical analyses

Mean ± standard deviation (SD) was used to describe the survival data of *G. mellonella*. Between-group comparisons were conducted using the log-rank test in SPSS v20.0 software. The survival curves were plotted using GraphPad Prism v9.3 software. For all tests, *P* ≤ 0.001 indicates a statistically significant difference.

## 3. Results

### 3.1. Patient clinical data

In July 2022, the patient, a 7-month-and-26-day-old infant male, presented at Shenzhen Maternal and Child Health Hospital with a fever peak of 38.3°C and two instances of non-jet vomiting with no obvious cause. The first and second days of admission, the patient received Cephalosporin antibiotics, along with fluid replacement, antipyretics, and other symptomatic treatments. However, recurrent fever persisted, and routine blood examinations showed an increase in C-reactive protein (CRP) to 110.51 mg/l, an elevation in procalcitonin from 0.46 ng/ml to 0.74 ng/ml, and an erythrocyte sedimentation rate of 90 mm/h. When a patient is suspected of having Bloodstream Infection upon admission to the pediatric ward, the standard procedure for sampling involves collecting blood samples from both sides of the patient's limbs at different time points. The blood culture reports indicated the presence of Gram-positive cocci, with the blood culture isolate identified as *M. luteus*. Cerebrospinal fluid analysis revealed no bacteria or fungi, excluding the possibility of intracranial infection. The patient's immunoglobulin levels are within the normal range, and there is no history of recurrent infections. After initiating treatment with Linezolid, the patient's body temperature returned to normal, and CRP levels decreased. The patient was diagnosed with: (i) bloodstream infection; (ii) acute gastroenteritis; (iii) acute upper respiratory tract infection; (iv) acute otitis media (bilateral); (v) mild anemia.

### 3.2. Pathogen identification

**Figure 1:**
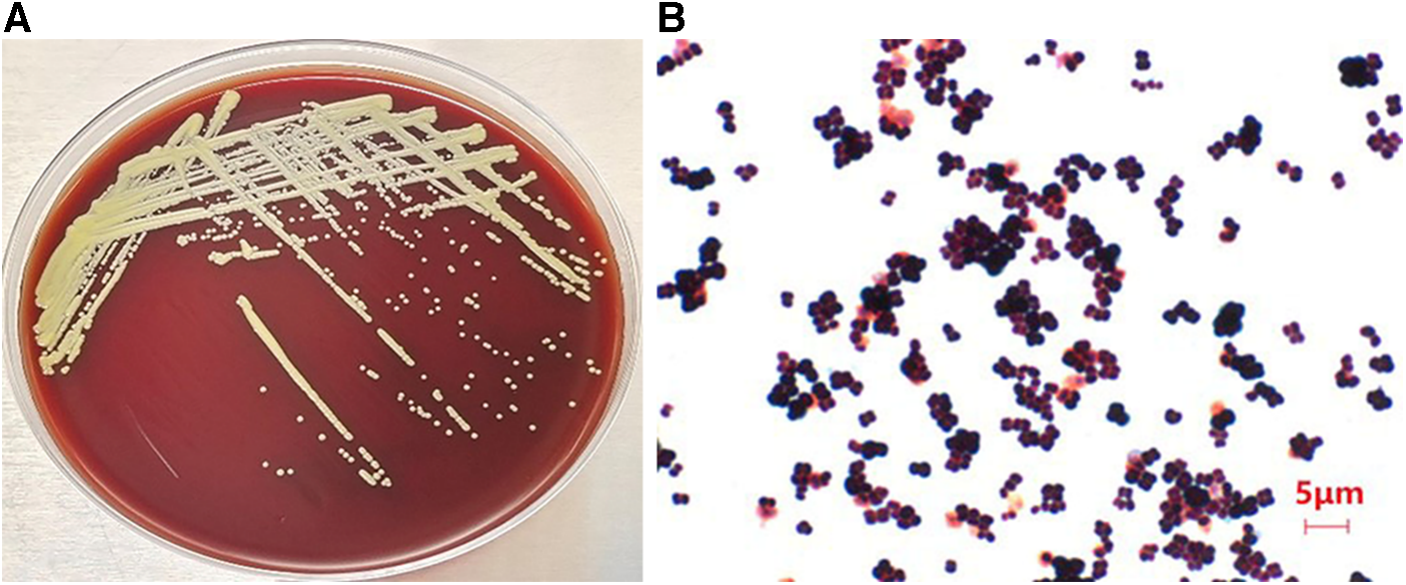
Images of isolated bacteria to depict morphology of (**A**) the colony on a blood plate and (**B**) gram positive cocci indicated by gram staining of a bacterial smear of picked colonies (×100 magnification).

Bacterial growth was relatively slow at 37°C over 24 h, and colony morphology was relatively small. After 48 h of incubation, colony size had increased to 1–2 mm and presented with a pale yellow, round and raised smooth surface with a neat edge (Figure 1A). Microscopic examination after Gram staining of bacterial smears showed Gram-positive cocci, most of which were arranged in pairs, fours, or clusters (Figure 1B). Mass spectrometry analysis identified the cocci to be *M. luteus* with a confidence percentage of 98.90%. The results of physicochemical characterization of the strains showed that the isolates of *M. luteus* were consistent with the results of reference in terms of colony color, growth temperature, motility, peroxidase, oxidase, mannose, xylose, lactose, mannitol, urea, galactose, and other physiological and biochemical characteristics (Table 1). The antibiotic susceptibility test showed that the isolated *M. luteus* strain was sensitive to 15 antibiotics, including Penicillin, Vancomycin, Erythromycin, Clindamycin, Moxifloxacin, Cefotaxime, Tetracycline, Linezolid, Cotrimoxazole, Meropenem, Chloramphenicol, Amoxicillin, Levofloxacin, Teicoplanin and Cefepime.

**Table 1:** Physiological and biochemical properties test results^a^.

Identification items	Reference standards^b^	Isolated strain^b^
Yellow	+	+
37°C growth	+	+
Sporty	–	–
Catalase	+	+
Oxidase	+	+
Mannose	–	–
Xylose	–	–
Lactose	–	–
Mannitol	–	–
Arginine	–	–
Urea	+	+
Galactose	–	–

^a^ For physiological and biochemical characteristics, refer to the *bergey’s manual of determinative bacteriology*.

^b^ “+” represents a positive result; “–” represents a negative result.

### 3.3. Genome analysis of the isolated strain

**Figure 2:**
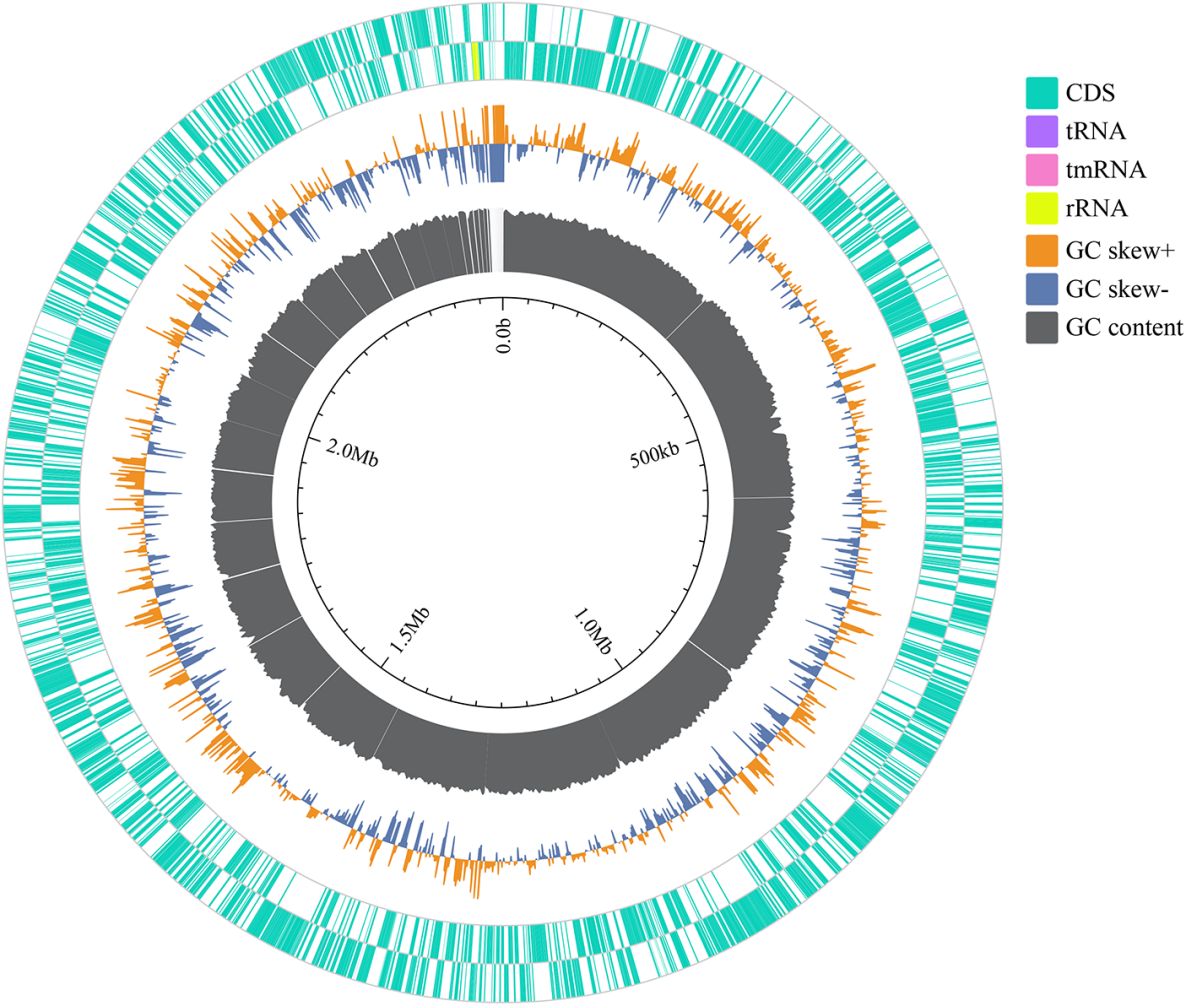
The genomic circular representation of the *Micrococcus luteus* isolate. From outside to inside, the various circles represent genes on coding DNA sequence (CDS green), RNA genes (tRNAs purple, tmRNAs pink, rRNAs yellow), GC skew, and GC content.

The genome was reassembled from scratch to obtain 57 sized contigs, with a total genome length of 2494966 bp and a guanine-cytosine (guanine-cytosine content, GC) content of 72.92%. Among the 2,291 predicted genes, 2,234 encode proteins, and 53 tRNAs, 1 tmRNA, and 3 rRNAs are also predicted (Figure 2). The results of species identification confirmed the bacterium to be *M. luteus*. Analysis of *M. luteus* virulence genes demonstrated a dominance of regulating amino acids and purine metabolism genes, as well as those involved in lipid and fatty acid metabolism, iron absorption, nutritional virulence, cell surface composition and type VI secretion system (T6SS) (Table 2). The commonly used ResFinder7 and CARD databases did not predict resistance genes.

**Table 2:** Virulence related genes the *Micrococcus luteus* isolate.

Virulence factors class	Virulence-associated genes
Adherence	*groEL, plr/gapA*
Enzyme	*eno*
Immune evasion	*rfbB-1*
Type VI secretion system	*clpB*
Amino acid and purine metabolism	*glnA1, leuD, lysA, purC*
Antiphagocytosis	*wbjD/wecB, wecC, gnd*
Cell surface components	*rmlA, kefB, sugA, sugB, sugC*
Iron uptake	*ideR, mbtI*
Lipid and fatty acid metabolism	*icl, panD*
Nutritional virulence	*carB*
Phagosome arresting	*ndk*
Protease	*pafA*
Regulation	*relA, phoP, regX3, sigA/rpoV, sigH*
Stress adaptation	*sodA*

### 3.4. Genomic analysis of blood pathogens

**Figure 3:**
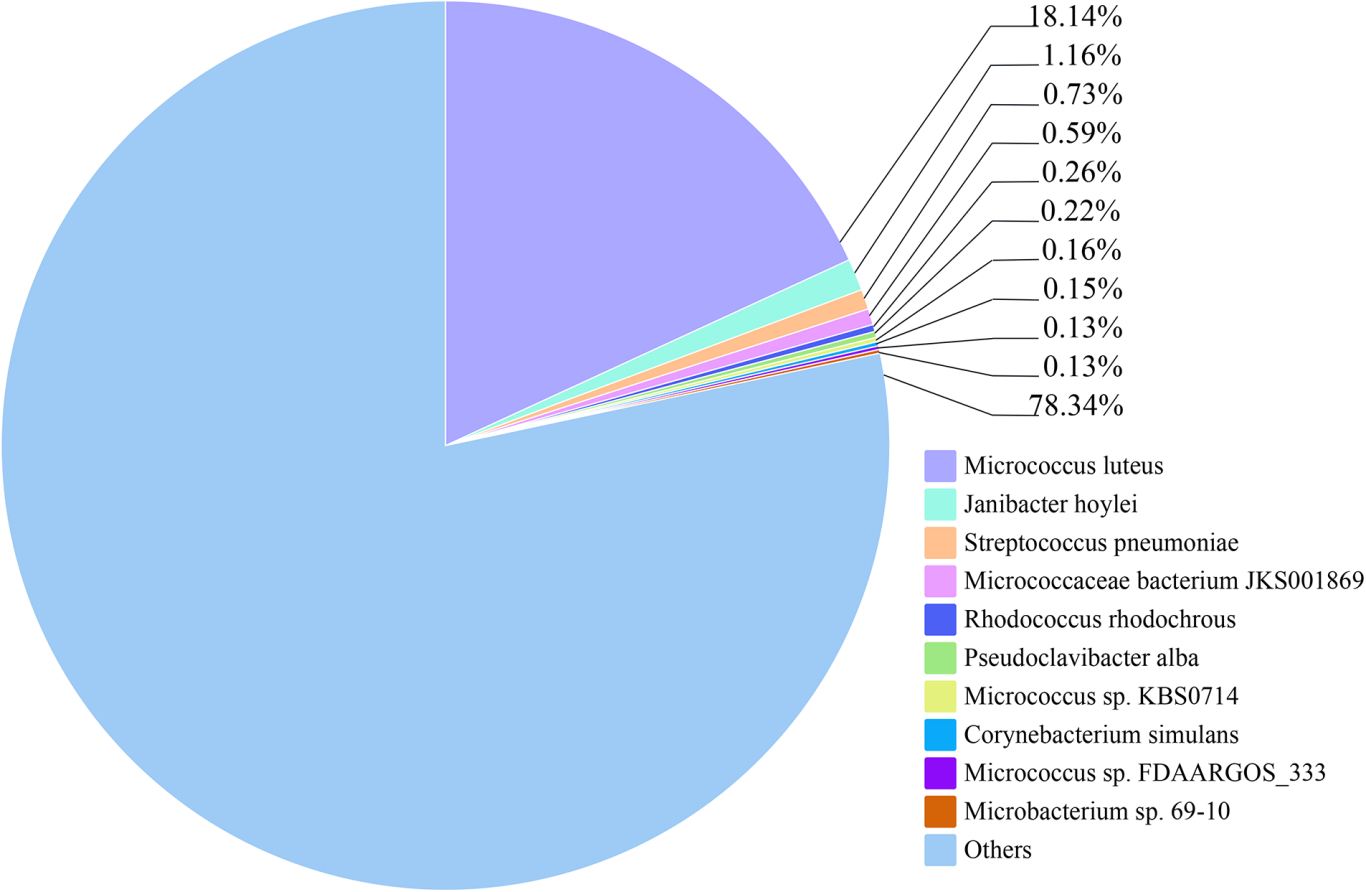
The relative abundance of the top 10 species of blood pathogens at the species level is reflected in the patient.

The relative abundance of species at different taxonomic levels, based on metagenomic data of blood pathogens, indicates that *M. luteus* contributed the highest proportion at 18.14%. *Janibacter hoylei, Streptococcus pneumoniae* and other *Micrococcus* spp., were also annotated by the LCA algorithm aside from the ‘Other pathogens’ category (Figure 3). ‘Others’ in the species richness pie chart indicates that the program could not predict the classification level according to the prescribed rules and information in the database.

### 3.5. Infection and survival of *Galleria mellonella* larvae

After 24 h inoculation with *M. luteus*, the *G. mellonella* larvae of the high dose group began to blacken and die. There was no larval mortality in the low and medium dose groups. The blank control group, puncture group, and physiological saline group showed no mortality of *G. mellonella* larvae, with a light-yellow appearance and sensitive response to external stimuli.

**Figure 4:**
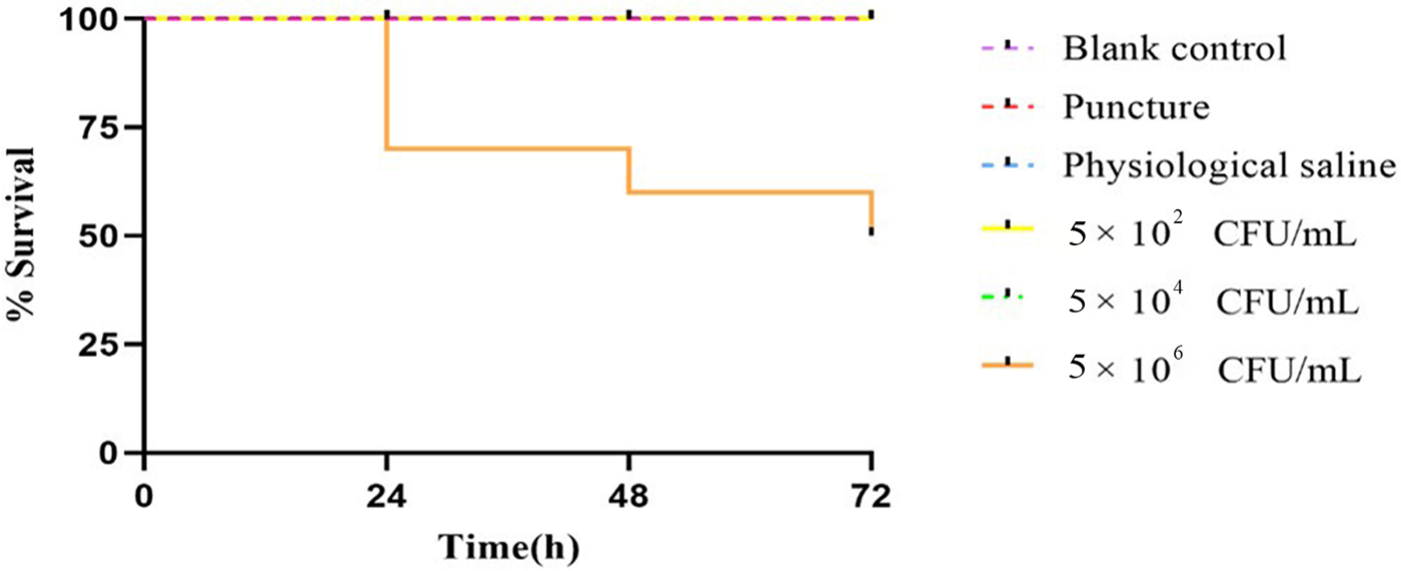
Survival of *Galleria mellonella* following infection by *Micrococcus luteus* strains. The data shown are means ± SD from three independent experiments recorded for 72 h. Differences in survival were calculated using the log-rank test for multiple comparisons. Differences were considered statistically significant at *P *< 0.001.

At 24 h post-inoculation, *G. mellonella* larvae survival rates in low, medium, and high dose groups of *M. luteus* isolate were 100% (10/10), 100% (10/10) and 70% (7/10) respectively. After 48 h of vaccination, the survival rates of low, medium, and high dose groups were 100% (10/10), 100% (10/10), and 60% (6/10), respectively; After 72 h of vaccination, the survival rates of the three groups were 100% (10/10), 100% (10/10), and 50% (5/10), respectively. There was a statistically significant difference in survival rates between the control group and the experimental group (*P* < 0.001), and there was a statistically significant difference in survival rates between low, medium, and high doses (*P* < 0.001) (Figure 4).

## 4. Discussion

*Micrococcus luteus* is an opportunistic Gram-positive coccus widely distributed in water, air, soil and other environments (1), which seldom was reported as human pathogenesis, but it could arose infection in some specific situation (3, 27). Currently, it is still challenging to distinguish between *M. luteus* infection and contamination. Clinical judgment, positive blood culture bottles, short time to growth period (time from bacterial inoculation to detection), and new microbiologic technologies are considered possible methods to avoid blood culture contamination (28). In this research, we were able to isolate *M. luteus* from positive blood cultures obtained from bilateral limbs at two different time points and identify a high concentration of *M. luteus* in the blood using metagenomics. Compare to blood culture contamination, the above method could further indicates that we found a blood infection caused by *M. luteus*. Considering the clinical information, the patient lacks a history of recurrent infections, and the immunoglobulin levels fall within the normal range, eliminating the possibility of hereditary immunocompromised disease. The presence of inflammation in the upper respiratory and gastrointestinal tracts, coupled with mild anemia and the young age of only 7 months and 26 days, renders the patient more susceptible to infections. Consequently, there is a likelihood of a lower immune level, creating an opportunity for opportunistic infection by *M. luteus*.

Effective treatment of *M. luteus* is important for people at increased risk of infection. There have been reports (28) indicating that Cephalosporins and Quinolones are effective empirical antibiotics for treating *M. luteus*. Vancomycin and Teicoplanin should be considered for potential extensively drug-resistant *M. luteus* strains. In this case, although antibiotic susceptibility testing showed that *M. luteus* was susceptible to Cephalosporins and other antibiotics, the patient continued to experience a fever and elevated inflammatory markers, such as C-reactive protein and procalcitonin, even after receiving cephalosporin treatment. Due to the possibility of rash in infant clinical treatment with Vancomycin, Linezolid, with a similar antibacterial spectrum to vancomycin, was eventually used and resulted in a decrease in body temperature. In summary, the empirically effective antibiotic, Cephalosporins, demonstrated inconsistency between its in antibiotic susceptibility test and actual clinical treatment outcomes. On the other hand, it's crucial to exercise caution regarding the adverse effects of Vancomycin in infant use. Ultimately, Linezolid proved to be an effective treatment for the infant. However, the research has its limitations. The incidence of bloodstream infections caused by *M. luteus* is low, and as of November 2023, only four articles related to *M. luteus* bloodstream infections were found on PubMed. There is limited research attention on *M. luteus* bloodstream infections, and consensus on empirical treatment for *M. luteus* infections has yet to be established. While the patient's clinical treatment implies the necessity of contemplating the potential for Cephalosporin resistance and the successful application of Linezolid in treating this infection, the limitation of having only one case emphasizes the need for additional case data on *M. luteus* bloodstream infections to support our conclusions. Furthermore, it is crucial to continue exploring the optimal treatment for *M. luteus* bloodstream infections.

*M. luteus*, a group of actinomycetes widely used in biotechnology, is considered as a new hospital pathogen (6, 29). Although the use of bioinformatics analysis could predict functionality, there is usually lack of validated pathogenicity to establish animal infection models that could help increase understanding of disease mechanisms (30, 31). At present, there are few reports on animal models of *M. luteus*. The results of the infection model of *M. luteus* larvae constructed in this study show that the larvae of *G. mellonella* can withstand low infection doses, generate immune responses against *M. luteus*, and kill the larvae of *G. mellonella* in a short time at high dose concentrations. The mortality and dose concentration are dependent, suggesting that *G. mellonella* larvae could be used as a model for the construction of *M. luteus* infection. The survival rate of bacteria could be used to judge the virulence of the strain, providing an important internal model selection for the infection of *M. luteus*. Based on the comprehensive clinical manifestations, although the patient experiences recurrent fever and elevated inflammatory markers, there are no clinical signs of sepsis. Moreover, *M. luteus* exhibits low toxicity when isolated from the blood of pediatric patients, as it can only kill larvae at high concentrations.

The virulence genes of the *M. luteus* are isolated in this study, including genes that regulate T6SS. T6SS is a nanomoleular complex, regulated by *clpB* gene, that can release virulence factors to target host cells (32) and exists widely in gram-negative bacteria. T6SS is involved in bacterial colonization, enhanced survival, adhesive modification, internalization and escape from the immune system (33) and can cause the destruction of lipid membrane and cytoskeleton (32). While we found the *clpB* gene of T6SS in the isolated strain of Gram positive coccus *M. luteus*, the potential role in bacterial virulence and human pathogenicity remains to be further explored.

## Data Availability

The datasets presented in this study can be found in online repositories. The names of the repository/repositories and accession number(s) can be found at: https://www.ncbi.nlm.nih.gov/sra/PRJNA973819, PRJNA973819.
